# The Effect of Increasing Nickel Content on the Microstructure, Hardness, and Corrosion Resistance of the CuFeTiZrNi*_x_* High-Entropy Alloys

**DOI:** 10.3390/ma15093098

**Published:** 2022-04-25

**Authors:** Po-Cheng Kuo, Sin-Yi Chen, William Yu, Ryo Okumura, Satoshi Iikubo, Andromeda Dwi Laksono, Yee-Wen Yen, Alberto S. Pasana

**Affiliations:** 1 Department of Materials Science and Engineering, National Taiwan University of Science and Technology, Taipei 10672, Taiwan; george807228@gmail.com (P.-C.K.); bobxxi.c@gmail.com (S.-Y.C.); m10504810@mail.ntust.edu.tw (W.Y.); andromeda@lecturer.itk.ac.id (A.D.L.); 2 Kyushu Institute of Technology, Graduate School of Life Science and Systems Engineering, Kitakyushu 808-0196, Japan; okumura.ryou278@mail.kyutech.jp (R.O.); iikubo.satoshi.472@m.kyushu-u.ac.jp (S.I.); 3 Department of Advanced Materials Science and Engineering, Kyushu University, 744 Motooka Nishi-ku, Fukuoka 819-0395, Japan; 4 School of Engineering, University of San Carlos (USC), Cebu City 6000, Philippines; aspasana16@gmail.com

**Keywords:** CuFeTiZrNi*_x_* alloys, microstructure, hardness, corrosion resistance, first-principles calculations

## Abstract

In recent years, high-entropy alloys (HEAs) that contain fine grains of intermetallic compounds (IMCs) have gained increasing attention as they have been shown to exhibit both high mechanical strength and strong corrosion resistance. One such class of HEAs is that of CuFeTiZrNi alloys. In this study, we have investigated the effect of increasing Ni content on the microstructure, hardness, and corrosion resistance of the CuFeTiZrNi*_x_* alloys (where *x* = 0.1, 0.3, 0.5, 0.8, 1.0 in a molar ratio). The alloys used in this study were prepared in an arc melting furnace and then annealed at 900 °C. First-principles calculations of the bulk modulus were also performed for each alloy. The results revealed that increasing the Ni content had several effects. Firstly, the microstructure of the CuFeTiZrNi*_x_* alloys changed from B2_BCC and Laves_C14 in the CuFeTiZrNi_0.1_ and CuFeTiZrNi_0.3_ alloys to FCC, B2_BCC, and Laves_C14 in the CuFeTiZrNi_0.5_ alloys; and to FCC, B2_BCC, Cu_51_Zr_14_, and Laves_C14 in the CuFeTiZrNi_0.8_ and CuFeTiZrNi_1.0_ alloys. Secondly, IMCs arising from a combination of the refractory elements (Ti and Zr) and atomic size differences were found in the interdendritic region. Thirdly, as the Ni content in the CuFeTiZrNi*_x_* alloys increased, the hardness decreased, but the corrosion resistance increased.

## 1. Introduction

The conventional approach to producing alloys with new microstructures and properties is to combine two principal elements in differing proportions. However, in 1995, Yeh et al. introduced a novel class of alloy called high-entropy alloys (HEAs) [1,2,3]. This proposal received much attention from academics and industries, especially those connected to metallurgy [4]. HEAs consist of five or more primary elements, each with an atomic concentration between 5% and 35%, mixed in equiatomic or near-equiatomic amounts [5,6,7]. In the years since HEAs were first proposed, many combinations of elements have been explored. These existing HEA systems serve as references for designing novel HEAs. The literature relating to these existing systems reports the fabrication of simple solid solutions, such as those with face-centered cubic (FCC), body-centered cubic (BCC), and hexagonal close-packed (HCP) structures [8], and discusses the effects on the properties of the HEAs of various physical parameters, such as the entropy of mixing (ΔS*_mix_*), enthalpy of mixing (ΔH*_mix_*), atomic size differences (δ), and parameter Ω that predicts the likelihood of forming a solid solution [9,10,11,12].

Several HEAs systems are reported in the literature, all of which have excellent properties suitable for a range of applications. In particular, many existing HEAs exhibit good mechanical strength, low thermal conductivity, electrochemical corrosion, and high-temperature softening resistance, as well as high thermal structural and chemical stabilities [5,8,13,14]. However, such properties are dependent on the chemical composition. The basic elements most often are Co, Cr, Fe, Ni, and Mn, while some other refractory elements are also employed, such as Ti, Ta, Mo, Nb, Al, and Zr [8,15,16,17]. With the addition of these latter elements, HEAs form not only with a single solid-solution phase but also with some intermetallic compounds (IMCs) in the system, such as µ, σ, and χ, and well-annealed HEAs comprise Laves phases [18]. Systems of this type, such as AlCoCrFe*_x_*Mo_0.5_Ni [5], Al_0.5_CoCrCuFeNi [13], AlCoCrCuFeNi [19], and Al_0.5_CoCrCuFeNiTi*_x_* [20], have been indicated as having many promising material properties.

Ye et al. have also reported the detection of many multi-phase HEAs [21]. Understanding how HEAs containing fine grains of an IMC form is important in developing future HEAs, as these phases can significantly affect the hardness of the alloys [8]. Furthermore, the effect of the Ni concentration on HEA alloys has been found by several researchers [22,23,24]. Increasing the nickel concentration from 1 to 1.8 can decrease the hardness of AlCrFeCoNi*x* alloys by approximately 43% to 316 HV because of the development of continuous solid solutions as a result of dissolution crystallites in a nickel-rich matrix [25], which is still higher than the SUS 304 with 265 HV [26]. The effect of the Ni concentration seems to need to be more investigated for better HEA properties. At the same time, forming IMC is rather challenging [27,28], especially in novel HEAs systems such as CuFeTiZrNi alloys. In this study, we have investigated the microstructure, hardness, and corrosion resistance of CuFeTiZrNi*_x_* alloys with increasing Ni content, with the aim of discovering a new high-performance HEA. We have also performed first-principles calculations to characterize the influence on the alloy structure of an increasing Ni content.

## 2. Experimental Procedure

### 2.1. Preparation of the CuFeTiZrNi_x_ Alloys and Analytical Method

High-purity (>99.9 at.%) Cu, Fe, Ti, and Zr elements were combined with varying amounts of Ni to form the CuFeTiZrNi*_x_* (where *x* = 0.1, 0.3, 0.5, 0.8, and 1.0 in a molar ratio) alloys. The total mass of the starting material for each alloy was 3.000 ± 0.001 g, and each alloy mixture was melted at least 5 times in an arc melting furnace (Miller, Gold Star 602; Appleton, WI, USA) to ensure that all elements mixed completely. Each specimen was then individually encapsulated in a quartz tube in a near-vacuum (below 0.1 N/m^2^) before being annealed at 900 °C for 2 h. After annealing, the specimens were cut into pieces and mounted in the Bakelite, and then given metallurgical treatment to ensure a smooth, defect-free surface. An optical microscope (OM; Olympus BX51M; Tokyo, Japan) and field emission-scanning electron microscope (FE-SEM; Jeol JSM-6500F; Tokyo, Japan) were used to examine the surface morphology and microstructure. The composition of each phase formed in the alloys was determined using SEM with an energy dispersive spectrometer (EDS; Oxford 7418; Oxford, UK) and an electron probe micro-analyzer (EPMA; Jeol JSM-8200; Osaka, Japan). Diffraction patterns for each region in the CuFeTiZrNi*_x_* alloys were generated using an X-ray diffractometer (XRD; D2 Phase Bruker; Ettlingen, Germany) with Cu-Kα radiation of 30 kV and 10 mA. A Vickers microhardness tester (HMV-2, Shimadzu; Kyoto, Japan) was employed to determine the Vickers Hardness number (HV) for each of the alloys, and the tests were carried out according to the regulation ASTM E92 [29], with each specimen subjected to 9.8 N (1 kg_f_) load for 15 s. Ten indentations were made on each alloy and the average value was calculated.

The Tafel extrapolation method was employed to the corrosion resistance of these five CuFeTiZrNi*_x_* alloys, with a Gamry Instruments system (Echem Analyst Software; Revision 1.2, Warminster, PA, USA) being used to measure the E*_corr_* (corrosion potential), *i_corr_* (corrosion current density), and CR (corrosion rate). The chemical test solution used was a 3.5 wt.% solution of NaCl. The Tafel measurements were performed at ambient temperature, in accordance with a previous study [30], and the potential range of the potentiodynamic polarization test was from −0.5 to 2 V with a scan rate of 1 mV/s.

For comparison with the Tafe method, a weight-loss test was applied based on the ASTM G31-72 laboratory standard [31]. In this test, the specimens were cut into pieces and then immersed in a 3.5 wt.% NaCl solution at ambient temperature for 30 days. After immersion, the corrosion rate (CR) was determined by the equation:

(1) $$\mathrm{Corrosion}\;\mathrm{Rate}\;(\mathrm{CR})=\frac{K\times W}{D\times A\times T}$$

where K is the corrosion constant (8.76 × 10^4^ mm/year), W is the weight loss, D is the density (g/cm^3^), A is the immersed surface area (cm^2^), and T is the immersion time (h). The specimen microstructure was examined by FE-SEM after the electrochemical test to determine the type of surface corrosion that had occurred.

### 2.2. First-Principles Calculations

The focus of this study is the CuFeTiZrNi*_x_* system, which is an alloy in which the five elements are mixed in approximately equimolar amounts. The physical properties of this CuFeTiZrNi*_x_* HEA system were ascertained with first-principles calculations using the Green function approach using Akai-KKP software [32]. This approach allows us to carry out the calculation for a disordered alloy. First, the formation energies of BCC and FCC solid solutions were calculated for 10,626 compositions in which the proportions of all 5 atoms were changed from 0.00 to 1.00 in 0.05 increments. Next, the effects of Ni content were investigated in more detail by determining the formation energies of BCC, FCC, and HCP solid solutions in which Fe, Cu, Ti, and Zr were equimolarized, and only the proportion of Ni was changed. In addition, the formation energy of the Laves phase of the binary system represented by AB_2_ (A = Ti, Zr, B = Cu, Fe, Ni) was obtained and compared with the calculation result of the solid-solution phase. Since the ratio of the atomic radius of the two atoms forming the Laves phase is 1.05–1.67, the atoms were selected to satisfy this condition. The Laves phase has a hexagonal C14 structure, C15 structure, and C36 structure. In this study, only the experimentally confirmed C14 structure was dealt with. In the structural optimization, a stable structure was obtained with an accuracy of 0.01 Bohr. However, in HCP, the axial ratio c/a of the lattice constant was fixed at the ideal ratio of 1.633. For the C14 Laves phase, the axial ratio was also changed to obtain a stable structure with an accuracy of 0.01 Bohr. Next, the Birch–Murnaghan equation of state was applied to fit the volume dependence of the energy and the hardness of the FCC, BCC, and Laves phase (C14). Finally, the results of the calculation were evaluated via comparison with the bulk modulus.

## 3. Results and Discussions

### 3.1. The Microstructure of the CuFeTiZrNi_x_ Alloys

**Figure 1:**
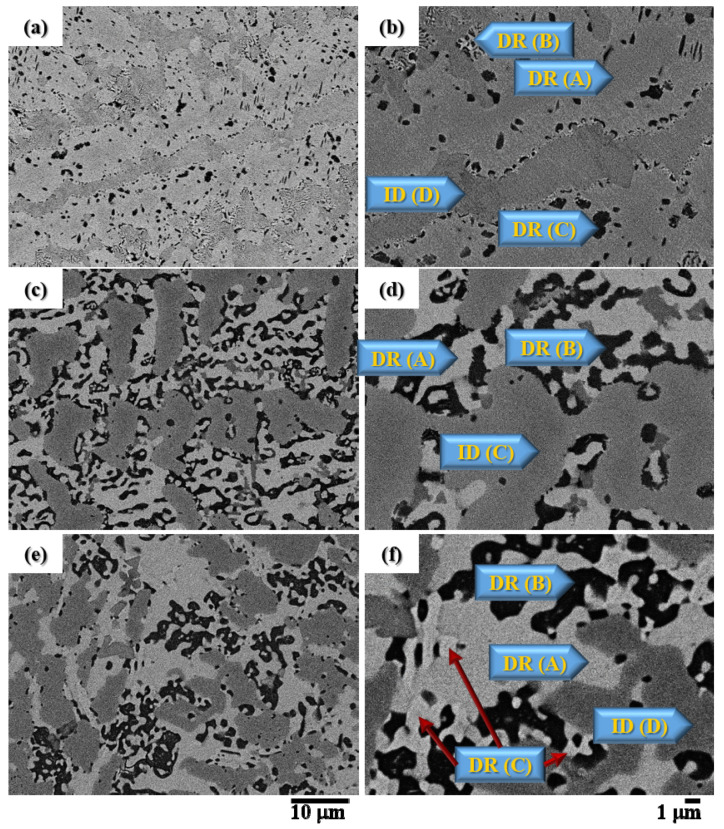
BEI microstructures of the CuFeTiZrNi*_x_* alloys with different nickel content(*x*): (**a**,**b**) *x* = 0.1, (**c**,**d**) *x* = 0.5, (**e**,**f**) *x* = 1.0 ((**a**,**c**,**e**) are at 2000× magnification and (**b**,**d**,**f**) are at 5000× magnification).

Figure 1 shows the back-scattered electron images (BEI) of the CuFeTiZrNi*_x_* alloys with *x* Figure 1a 0.1, Figure 1c 0.5, and Figure 1e 1.0; Figure 1b,d,f shows magnified images of Figure 1a,c,e. Two Dendrite (DR) and Interdendrite (ID) regions comprised of the solid solutions of the B2_BCC and FCC phases and intermetallic compounds (IMCs) of the Cu_51_Zr_14_ and Laves_C14 phases were observed. The composition of each phase is listed in Table 1. The Cu_51_Zr_14_ phase was only observed when *x* = 0.8 and 1.0. The Laves_C14 phases have a stoichiometry of AB_2_ and are normally formed when there is a large size difference between constituent atoms [33].

The mixing enthalpy (∆H*_mix_*), mixing entropy (∆S*_mix_*), atomic radius difference (δ), valence electron concentration (VEC) and Ω [12] of the five CuFeTiZrNi*_x_* alloys were calculated and are listed in Table 2. It was found that the ideal criteria for forming HEAs’ solid solution phases (FCC, BCC, and their mixtures including both ordered and disordered cases) were −22 ≤ ∆H*_mix_* ≤ 7 kJ/mol, 11 ≤ ∆S*_mi_*_x_ ≤ 19.5 J/mol K, δ ≤ 8.5, and Ω ≥ 1 [34,35,36,37,38,39]. However, the δ and Ω values of all the CuFeTiZrNi*_x_* alloys were found to be larger than 10 and 1.0, respectively.

**Figure 2:**
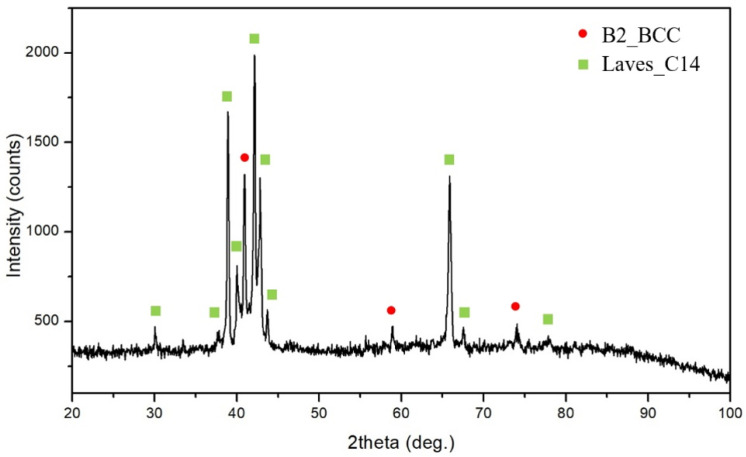
XRD pattern of the CuFeTiZrNi_0.1_ alloy.

The microstructure of the CuFeTiZrNi_0.1_ alloy has four distinct regions labeled A, B, C, and D in Figure 1b. The (A) light-grey and (B) white DR regions of the CuFeTiZrNi_0.1_ alloy were determined to be the Laves_C14 phase with crystal properties: Hexagonal structure, Pearson symbol-hP12, Space group-P6_3_/mmc, and lattice parameters of a = 5.130 Å, and c = 8.250 Å. This Laves_C14 phase in the CuFeTiZrNi_0.1_ alloy was referred to as the Cu_2_TiZr phase and labeled as the (Cu,Fe,Ni)_2_TiZr phase. In this alloy, the Cu, Fe, and Ni atoms can substitute each other because they have the same structure and their atomic size and electronegativity are similar [33]. The DR (C) region, composed of considerable concentractions of Ti and Fe (dark in contrast), was of the B2_BCC phase and was referred to as the FeTi-type and labeled as the (Fe,Ni)Ti phase. The ID (D) region (dark grey) was also identified as a Laves_C14 phase (hexagonal structure, Pearson symbol: hP12, Space group: P6_3_/mmc, a = 4.962 Å, c = 16.150 Å). It was referred to as the Fe_2_Zr phase and labeled the (Fe,Cu)_2_Zr phase. The composition of these phases in each alloy is listed in Table 1. Figure 2 shows the XRD pattern of the CuFeTiZrNi_0.1_ alloy. Peaks corresponding to the B2_BCC and Laves_C14 phases were found. It can be seen that the XRD result and compositional analyses are consistent with one another. In a typical alloy system, the Cu is restricted to the Dendrite (DR) region, which can be explained as being due to the bonding energy between Cu and other elements [19,24]. When the molar ratio of Ni reached 0.3, the phase at each region was seen to remain unchanged.

**Figure 3:**
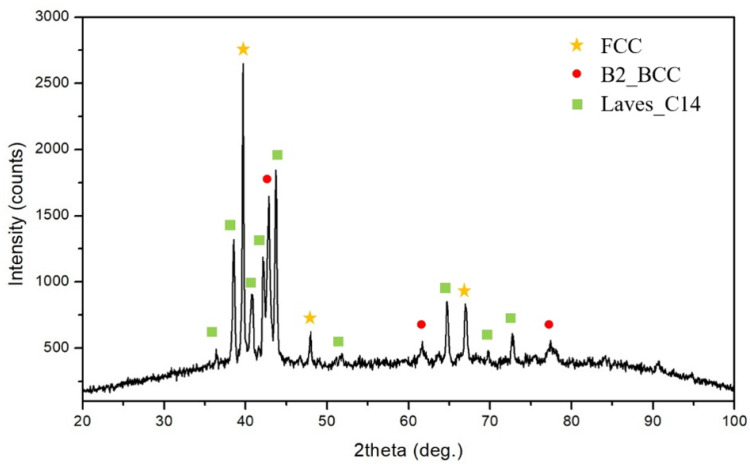
XRD pattern of the CuFeTiZrNi_0.5_ alloy.

The XRD pattern of the CuFeTiZrNi_0.5_ alloy is shown in Figure 3. The characteristic peaks corresponding to the FCC, B2_BCC, and Laves_C14 phases were found. Figure 1c,d shows the alloy’s microstructure. In Figure 1d, the light grey region was labeled as DR (A); it was an FCC phase (Cu-rih, Pearson symbol: cF4, and Space group: Fm $\bar{3}$ m). The dark region was labeled DR (B) and it was a B2_BCC phase, which was referred to as the FeTi-type and labeled as the (Fe,Ni)Ti phase with a = 3.015 Å. This B2_BCC phase tended to form a spinodal structure as the alloy cooled [19]. Spinodal decomposition usually occurs when a system has at least one pair of atoms with a positive enthalpy of mixing [24,35]. The ID (C) (dark grey) region was a Laves_C14 phase (hexagonal structure, Pearson symbol: hP12, Space group: P6_3_/mmc, lattice parameter: a = 4.962 Å and c = 16.15 Å); it was referred to as the Fe_2_Zr phase and labeled as the (Cu,Fe)_2_Zr phase. The detailed composition of each region is listed in Table 1. When the Ni content reached 0.5 in the molar ratio, Cu tended to bind with Ni to form an FCC phase due to the increase in Ni content increasing the electronegativity of Cu. This result indicated that this greater electronegativity had a stronger effect than the combined effect: (i) Increased mixing enthalpy, (ii) increased mixing entropy, and (iii) a minimal difference in the atomic radius [36].

**Figure 4:**
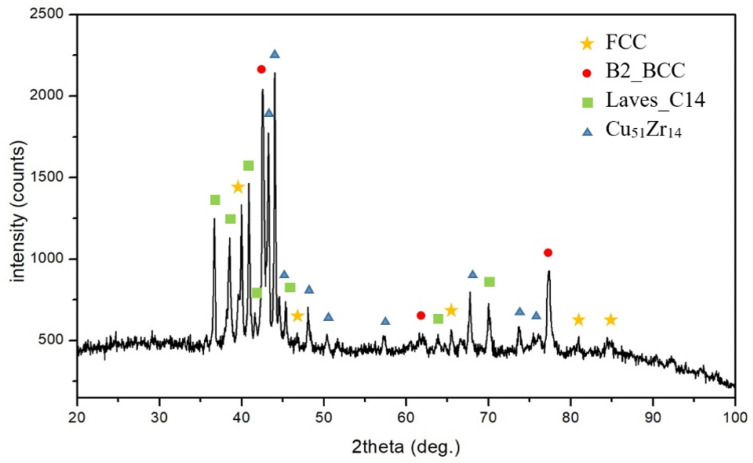
XRD pattern of the CuFeTiZrNi_1.0_ alloy.

As shown in Figure 4, the characteristic peaks of the FCC, B2_BCC, Laves_C14, and Cu_51_Zr_14_ phases were observed in the XRD pattern of the CuFeTiZrNi_1.0_ alloy. The microstructure of this alloy is shown in Figure 1e,f. Similar results for the XRD pattern and microstructure were found for the CuFeTiZrNi_0.8_ alloy. The microstructure of the CuFeTiZrNi_1.0_ alloy could be summarized as follows: (1) The DR (A) (light grey region) was an FCC phase (Cu-rich, Pearson symbol: cF4, and Space group: Fm $\bar{3}$ m); (2) the DR (B) (dark contrast region) was the B2_BCC phase (referred to as the FeTi-type and labeled as (Fe,Ni)Ti, a = 3.014 Å); (3) the white region-DR (C) was the Cu_51_Zr_14_ phase, which was the intermetallic compound (IMC) (hexagonal structure, Pearson symbol: hP65, Space group: P6/m, lattice parameter: a = 11.2348 Å, and c = 8.2708 Å); and (4) the ID (D) (dark grey region) was the Laves_C14 phase (hexagonal structure, Pearson symbol: hP12, Space group: P6_3_/mmc, lattice parameter: a = 4.962 Å, and c = 16.15 Å) referred to as the Fe_2_Zr phase and labeled as the (Cu,Fe)_2_Zr phase. The evolution and composition of all the phases formed in the CuFeTiZrNi*_x_* alloys are listed in Table 1. Overall, the addition of a larger molar amount of Ni to CuFeTiZrNi*_x_* alloy tended to form the FCC phase. This finding indicates that the Ni acts as an FCC stabilizer.

### 3.2. The Hardness Values of the CuFeTiZrNi_x_ Alloys

**Figure 5:**
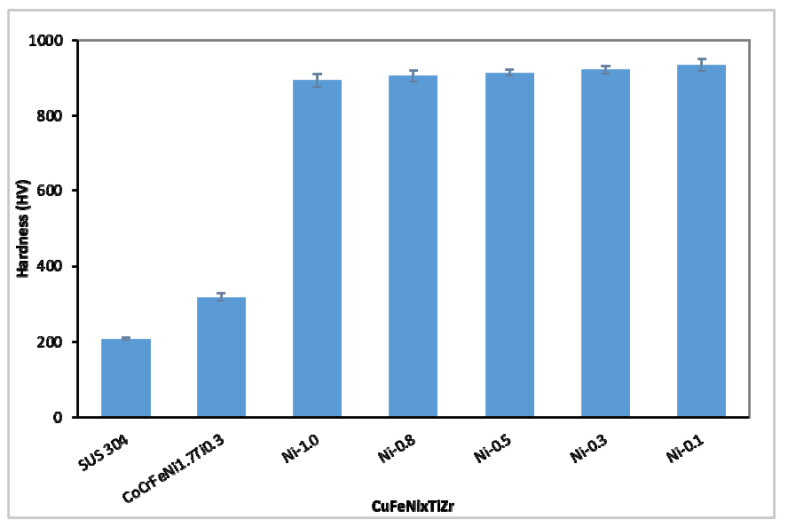
The hardness (HV) values of the CuFeTiZrNi*x* alloys, CoCrFeNi1.7Ti_0.3_ [34], and SUS 304.

HEAs and CCAs (complex concentrated alloys) of differing compositions differ significantly in hardness values. This is due to three critical factors: (1) The hardness, (2) relative volume ratio, and (3) morphology of each of the phases of which the alloys are composed [37]. Figure 5 and Table 3 show the hardness values (HV) of the CuFeTiZrNi*_x_* alloys, and also CoCrFeNi_1.7_Ti_0.3_ [38], and stainless steel 304 (SUS 304). It can be seen that the hardness values of the CuFeTiZrNi*_x_* alloys were high but also very different from those of both CoCrFeNi_1.7_Ti_0.3_ and SUS 304. This is likely to be due to the CuFeTiZrNi*_x_* alloys containing the Laves_C14 or IMC-Cu_51_Zr_14_ phases. The hardness of these two phases is higher than that of the BCC and FCC phases [39]. The maximum hardness was found in the CuFeTiZrNi_0.1_ alloy system and the value was 934.8 ± 17.0 HV. Increasing the Ni content in the CuFeTiZrNi*_x_* alloys results in more of the FCC phase forming in the alloys. As the FCC phase has low hardness, this results in the hardness of the alloy gradually decreasing with increasing Ni content.

### 3.3. First-Principles Calculation for the Hardness Properties of the CuFeTiZrNi_x_ Alloys

**Figure 6:**
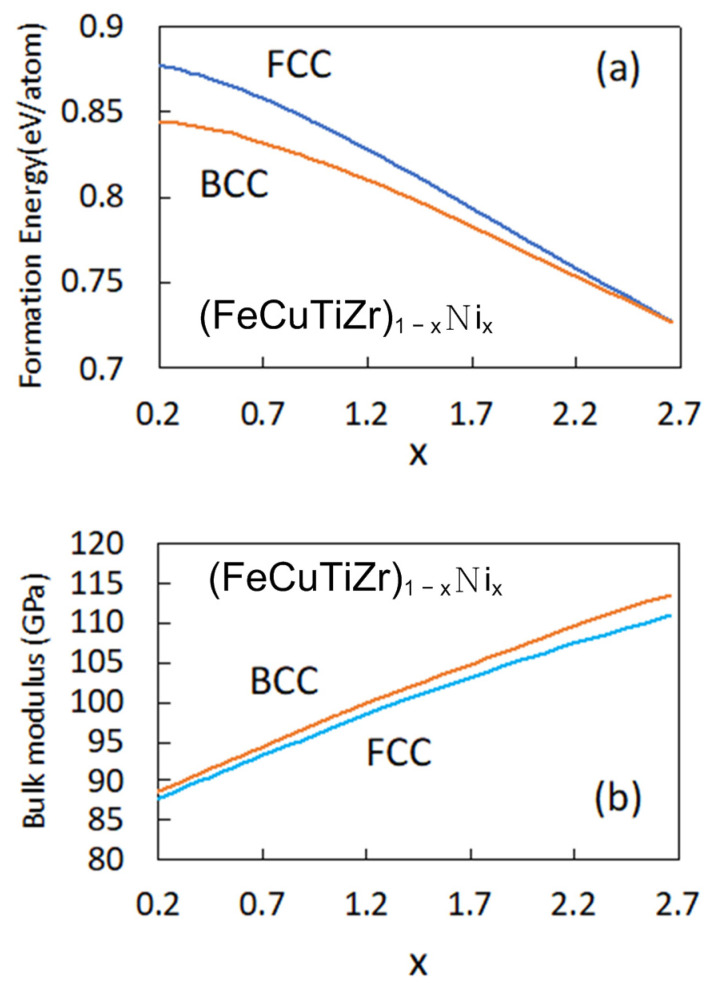
The dependence on Ni content of (**a**) the formation energies and (**b**) the bulk modulus of the FCC and BCC phases.

Figure 6 shows the calculation results for the formation energy (Figure 6a) and bulk modulus (Figure 6b) of HEAs with equimolar amounts of Fe, Cu, Ti, and Zr, and increasing Ni content. The calculated concentration range is from 5 to 40 at.% Ni in 1 at.% increments and the amount of Ni is denoted by *x* in Figure 6. It can be seen from Figure 6a that the BCC phase is more stable than the FCC phase when the Ni content is increased. In addition, the difference in formation energy between BCC and FCC phases decreases as *x* increases, and when *x* = 2.67 (Ni concentration is 40 at.%), the formation energy of the FCC phase is lower than that of the BCC phase. In other words, the formation of the FCC phase became more stable than that of the BCC phase when the value of *x* was greater than 2.67. In the experimental observations, only the Laves phase and the BCC phase were found when at *x* = 0.1 or 0.3, but the FCC phase was found when *x* = 0.5, 0.8, and 1.0. In addition, it was found that the proportion of BCC and FCC phases increased and the proportion of the Laves phase decreased as the Ni concentration increased.

Figure 6b shows the bulk modulus of FCC and BCC phases versus the molar ratio of Ni. The bulk modulus *B*_0_ is $B_{0}=V_{0}(\partial P/\partial V)$, where *V*_0_ is the volume at the ground state, *P* is the pressure, and *V* is the volume. This bulk modulus relates to changes in volume stress and is a function of the strength of the FCC and BCC phases. It can be seen from Figure 6b that the BCC phase has a slightly higher bulk modulus than the FCC phase. Figure 6b also confirms that the bulk modulus was increased with the increase in *x*. In the experimental observation, it was seen that the hardness of the CuFeTiZrNi*_x_* alloys decreased with increasing Ni concentration. This result is inconsistent with the calculation result that the bulk modulus increases with increasing Ni concentration. In the CuFeTiZrNi*_x_* HEA alloys, the bulk modulus of the solid solution phase on hardness might be considered to have only a small effect on the alloy hardness.

**Figure 7:**
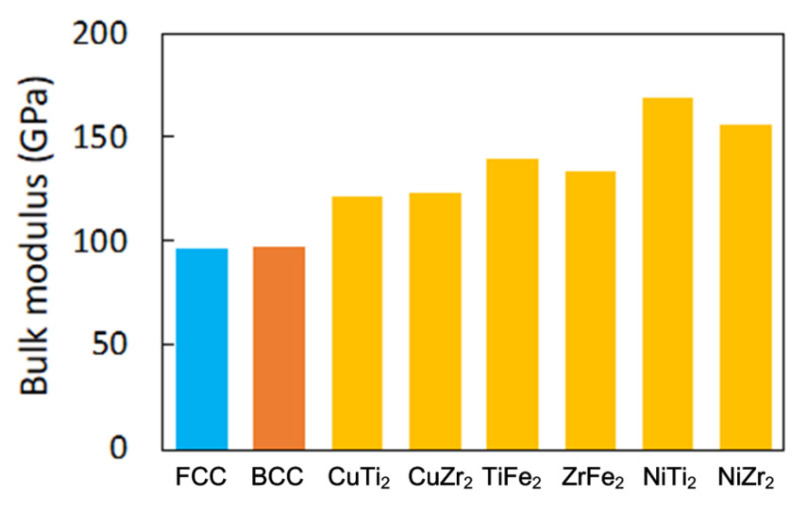
The bulk modulus of the FCC, BCC, and C14 binary Laves phases in the CuFeTiZrNi*_x_* alloy.

Figure 7 shows the calculated results for the bulk modulus of the Laves_C14 phase for the TiCu_2_, ZrCu_2_, TiFe_2_, ZrFe_2_, TiNi_2_, and ZrNi_2_ phases compared with the FCC and BCC phases in the equimolar FeCuTiZrNi*_x_* alloy. As shown in Figure 7, the six Laves_C14 phases show a higher bulk modulus than that in the FCC and BCC phases. The experimentally observed high hardness in the CuFeTiZrNi*_x_* HEA is attributed to the presence of the Laves_C14 phase with a high bulk modulus, and the decrease in hardness with the increase in the Ni concentration results in the increase in FCC and BCC phases with a low bulk modulus. In other words, when the Ni content in the CuFeTiZrNi*_x_* alloys was increased, more of the FCC phase was formed in CuFeTiZrNi*_x_* alloys. The intermetallic compound is harder for the metallic system than the single-solution phase. Thus, more of the FCC phase forming in the CuFeTiZrNi*_x_* alloys leads to a decrease in hardness. These statements are confirmed by other references that suggest that due to its solid solution or lack of an ordered phase and lower density, alloys containing an FCC phase have a reduced hardness [40]. Otherwise, the hardness would increase when the content of the FCC phase decreases because of precipitation hardening [41]. The first-principles calculations reveal that the Laves_C14 phase exhibits a higher bulk modulus than FCC and BCC and that the FCC phase is increasingly stable as the Ni concentration increases. In light of these results, it revealed that a computational study is an effective approach to predicting structural stability and hardness trends.

### 3.4. The Corrosion Resistance of the CuFeTiZrNi_x_ Alloys

**Figure 8:**
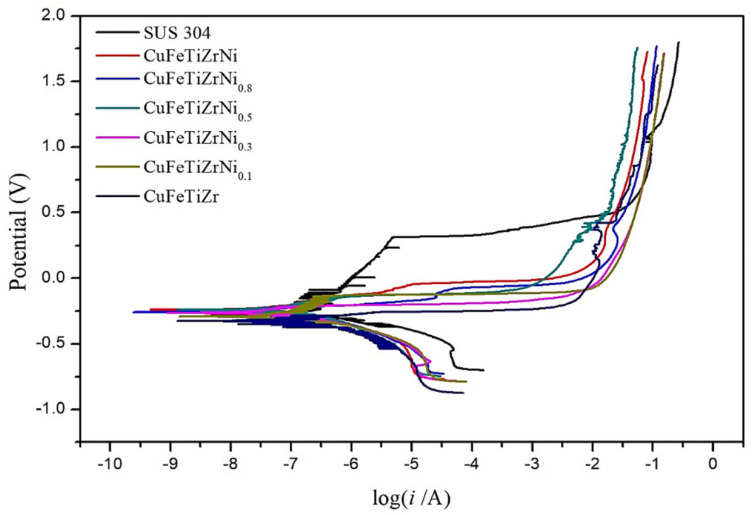
The polarization curves for the CuFeTiZrNi*_x_* alloys, CuFeTiZr [36], and SUS304 in 3.5 wt.% NaCl solution.

Figure 8 shows the polarization curves obtained from the CuFeTiZrNi*_x_* alloys immersed in a 3.5 wt.% solution of NaCl. It can be seen from Figure 8 that the addition of Ni improved the corrosion-resistant properties of the CuFeTiZrNi*_x_* alloys in a 3.5 wt.% NaCl solution compared with those of two references: The CuFeTiZr alloy [42] and SUS 304. In particular, the corrosion potential of the CuFeTiZrNi*_x_* alloys with higher Ni content (*x* = 0.5 to 1.0) was slightly higher than that of SUS304. This phenomenon revealed that the increase in Ni and decrease in Cu in the CuFeTiZrNi*_x_* alloys led to the formation of a Ni oxide layer. The Ni oxide layer acts as a protective layer to prevent damage to the surface of the CuFeTiZrNi*_x_* alloys. It is also reported in the literature that Cu in the alloys can damage this protective oxide film via a chemical reaction, for example, between Cu and the Cl of the NaCl solution, which would lead to the formation of holes or defects on the surface of the alloy [43,44]. Thus, the passivation behavior in the polarization curves of the CuFeTiZrNi*_x_* alloys in the 3.5 wt.% NaCl solution occurred at an anodic branch. This passive layer protected against corrosion and decreased the rate of corrosion of the CuFeTiZrNi*_x_* alloys [45]. The corrosion current (*i_corr_*) and corrosion voltage (E*_corr_*) values of the CuFeTiZrNi*_x_* alloys immersed in the 3.5 wt.% NaCl solution were also compared with those of the CuFeTiZr [42], SUS304, and CoCrFeNi_1.7_Ti_0.3_ [38] systems. The values are listed in Table 4 and show trends consistent with those of the polarization curves presented in Figure 8.

**Figure 9:**
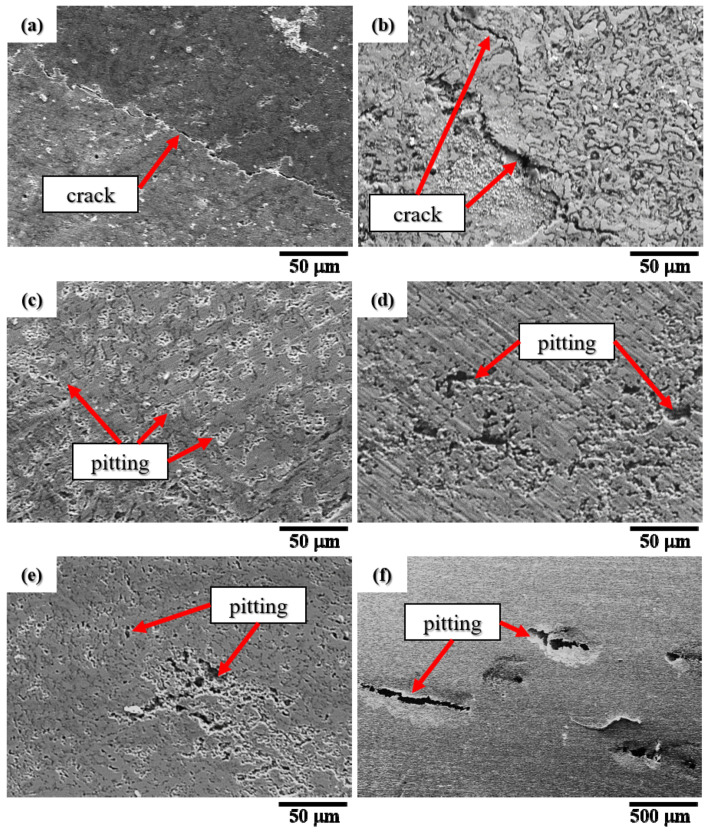
SEI surface morphologies of the CuFeTiZrNi*_x_* alloys after the polarization test in 3.5 wt.% NaCl solution: (**a**) *x* = 0.1, (**b**) *x* = 0.3, (**c**) *x* = 0.5, (**d**) *x* = 0.8, (**e**) *x* = 1.0, and (**f**) SUS 304.

Figure 9 show the secondary electron image (SEI) micrographs of the CuFeTiZrNi_0.1_ in Figure 9a, CuFeTiZrNi_0.3_ in Figure 9b, CuFeTiZrNi_0.5_ in Figure 9c, CuFeTiZrNi_0.8_ in Figure 9d, CuFeTiZrNi_1.0_ in Figure 9e, and SUS 304 in Figure 9f after the polarization test. Stress corrosion creak (SCC) was observed on the surfaces of the CuFeTiZrNi_0.1_ and CuFeTiZrNi_0.3_ alloys, as shown in Figure 9a,b. This SCC was due to the formation of a protective oxide or passivation film on the surface. As a result, the surface of the alloy was not directly exposed to the 3.5 wt.% NaCl solution. Sodium ions generated by the electrolyte exchange electrons within the reaction environment [42]. According to the literature, SCC arises from a combination of the presence of tensile stress and the specific corrosion medium leading to hydrogen embrittlement. When SCC has occurred, the metal remains intact over most of its surface while fine cracks progress through it. This SCC exhibits a brittle mechanical fracture [46]. In addition, as shown in Figure 9c–f, pitting corrosion was observed on the surface at the passivation area in both the CuFeTiZrNi*_x_* (*x* = 0.5, 0.8, and 1.0) alloys and SUS 304. Pitting represents a highly localized attack that results in holes in the alloy. The degree of pitting decreased with increasing Ni content, and the petting cavities were smaller than those observed in the SUS 304 substrate.

**Figure 10:**
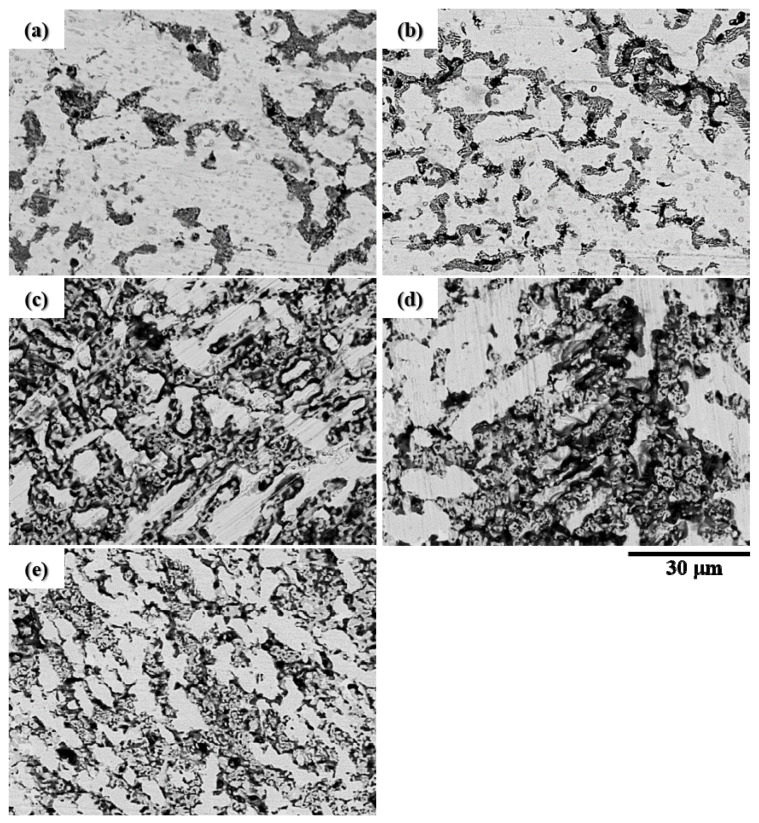
BEI surface morphologies of the CuFeTiZrNi*_x_* alloys after the polarization test in 3.5 wt.% NaCl solution: (**a**) *x* = 0.1, (**b**) *x* = 0.3, (**c**) *x* = 0.5, (**d**) *x* = 0.8, and (**e**) *x* = 1.0.

Figure 10 shows the BEI morphologies of all the CuFeTiZrNi*_x_* alloys following the polarization test. It can be seen that the Cu-rich regions were selectively etched while the other regions were undamaged. This shows that the other regions were relatively stable. This phenomenon results from differences in the chemical composition across the surface of the alloy. Under the action of the corrosive medium, the active region was oxidized while the other regions remained stable. This Cu-rich region corroded because the Cu atom is highly reactive and readily forms oxide. However, the resulting Cu oxide layer cannot provide good corrosion protection.

**Figure 11:**
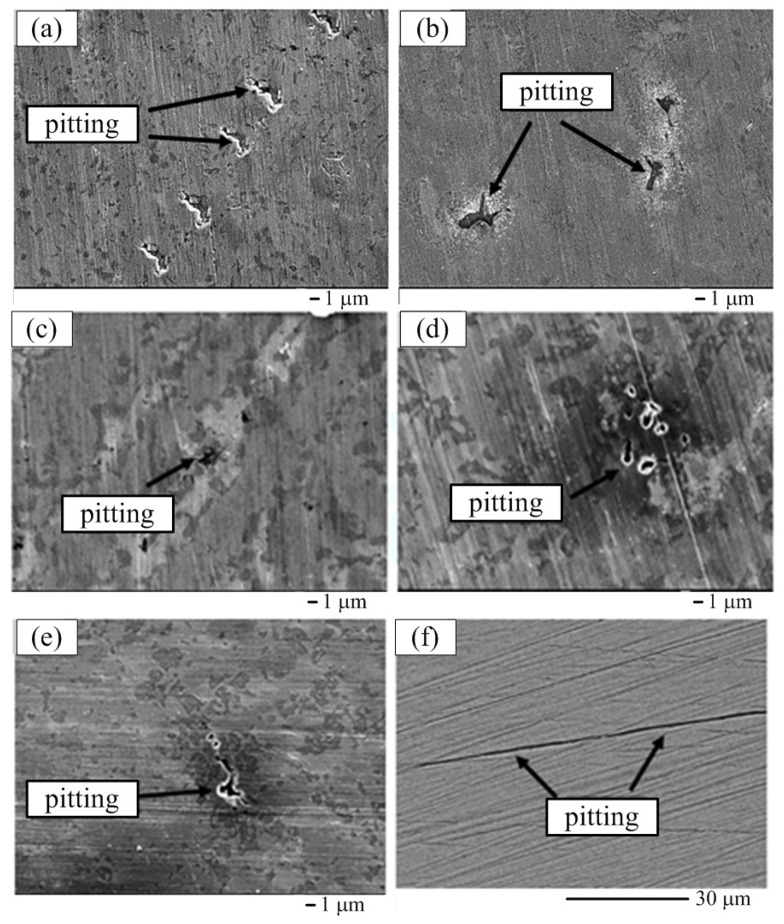
SEI surface morphologies of the CuFeTiZrNi*_x_* alloys after the polarization test in 3.5 wt.% NaCl solution for 30 days: (**a**) *x* = 0.1, (**b**) *x* = 0.3, (**c**) *x* = 0.5, (**d**) *x* = 0.8, and (**e**) *x* = 1.0, and (**f**) SUS 304.

Figure 11 shows the SEI surface morphologies of the CuFeTiZrNi*_x_* alloys and SUS 304 after being immersed in the 3.5 wt.% NaCl solution for 30 days. The main type of corrosion behavior observed was surface pitting of the Cu-rich region in the CuFeTiZrNi*_x_* alloys. However, the pitting types between CuFeTiZrNi*_x_* alloys and SUS 304 differed, with cavities being observed in the CuFeTiZrNi*_x_* alloys while a knife-line attack (KLA) was seen to form in the SUS 304. This difference arose from the stabilized austenitic stainless steel being attacked intergranularly by chromium carbide precipitation [46].

**Figure 12:**
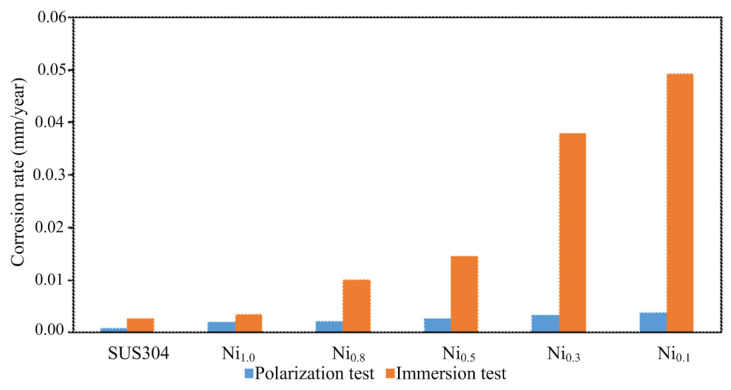
A comparison of the polarization and immersion test results of the CuFeTiZrNi*_x_* alloys and SUS 304.

The average corrosion rates of the CuFeTiZrNi*_x_* alloys and SUS 304 from the polarization and immersion tests are listed in Table 5 and plotted in Figure 12. The corrosion rate obtained from the weight loss data was used only as an index of the intensity of the corrosion attack [47]. The result revealed that the corrosion rate decreased slightly with an increasing Ni content due to the corresponding decrease in Cu content. The CuFeTiZrNi*_x_* alloys corroded faster than the SUS 304 because the SUS 304 contained no Cu. Cu did not generate a good protective oxide film on the surface of the alloy. The results confirmed that having more Ni and less Cu slowed down the corrosion of the surface of the CuFeTiZrNi*_x_* alloy.

## 4. Conclusions

In this study, the microstructure, hardness, and corrosion resistance of CuFeTiZrNi*_x_* high-entropy alloys were investigated. The following conclusions could be drawn.

1. The B2_BCC and Laves_C14 phases were found in all CuFeTiZrNi*_x_* alloys. When *x* was greater than 0.5, the FCC phase was also formed. When *x* increased to 0.8 and 1.0, the Cu_51_Zr_14_ phase was also observed in the CuFeTiZrNi*_x_* alloys.
2. The hardness of the CuFeTiZrNi*_x_* alloys gradually decreased with increasing Ni content. More of the FCC phase formed in the CuFeTiZrNi*_x_* alloys as the Ni content increased, and, as shown by first-principles calculations of hardness, the FCC phase had the lowest bulk modulus. Thus, more of the FCC phase forming in the CuFeTiZrNi*_x_* alloys leads to decreased hardness.
3. The corrosion resistance properties of the CuFeTiZrNi_0.5_, CuFeTiZrNi_0.8_, and CuFeTiZrNi_1.0_ alloys were superior to those of the SUS 304 and CoCrFeNi_1.7_Ti_0.3_ alloy systems in 3.5 wt.% NaCl solution. An increase in Ni content and the corresponding decrease in Cu content improved the corrosion potential and decreased the corrosion current density, indicating a gradual enhancement in corrosion resistance.
4. In the polarization test, stress corrosion creaking (SCC) was observed in the CuFeTiZrNi_0.1–0.3_ alloys, as well as pitting in the CuFeTiZrNi_0.5–1.0_ alloys. The corrosion rates in both the polarization and immersion tests decreased slightly with increasing Ni content. In the immersion test, the major type in all CuFeTiZrNi*_x_* alloys was surface pitting.

## Figures and Tables

**Table 1:** The chemical composition of the CuFeTiZrNi*_x_* alloys in terms of atomic percentage (*x* = 0.1, 0.3, 0.5, 0.8, and 1.0 in molar ratio).

Alloys	Structure	Phase	Phase Composition (at.%)				
			Cu	Fe	Ni	Ti	Zr
CuFeTiZrNi_0.1_	Dendrite-A	Laves_C14	32.5	17.2	2.0	23.8	24.5
	Dendrite-B	Laves_C14	38.0	14.6	4.0	20.2	23.2
	Dendrite-C	B2_BCC	18.5	32.5	2.6	38.5	7.9
	Interdendrite-D	Laves_C14	25.8	27.4	2.0	21.2	23.6
CuFeTiZrNi_0.3_	Dendrite-A	Laves_C14	29.2	16.3	8.0	24.3	22.2
	Dendrite-B	Laves_C14	33.0	16.2	8.5	20.7	21.6
	Dendrite-C	B2_BCC	20.6	25.5	6.4	35.0	12.5
	Interdendrite-D	Laves_C14	26.0	25.2	6.2	19.1	23.5
CuFeTiZrNi_0.5_	Dendrite-A	FCC	43.4	4.3	14.0	9.5	28.8
	Dendrite-B	B2_BCC	15.8	25.3	9.8	40.2	8.9
	Interdendrite-C	Laves_C14	17.4	33.0	7.5	20.6	21.5
CuFeTiZrNi_0.8_	Dendrite-A	FCC	35.2	3.1	23.1	10.8	27.8
	Dendrite-B	B2_BCC	13.6	21.1	17.9	38.9	8.5
	Dendrite-C	Cu_51_Zr_14_	51.4	10.0	11.6	10.3	16.7
	Interdendrite-D	Laves_C14	15.7	34.5	10.9	18.9	20.0
CuFeTiZrNi_1.0_	Dendrite-A	FCC	31.0	3.0	28.3	12.6	25.1
	Dendrite-B	B2_BCC	11.2	22.3	19.7	40.0	6.8
	Dendrite-C	Cu_51_Zr_14_	55.1	6.9	12.0	8.3	17.7
	Interdendrite-D	Laves_C14	13.6	35.6	14.2	17.9	18.7

**Table 2:** ∆H*_mix_*, ∆S*_mix_*, δ, VEC, and Ω values for the CuFeTiZrNi*_x_* alloys.

Alloy	∆H*_mix_* (kJ/mol)	∆S*_mix_* (J/K mol)	δ	VEC	Ω
CuFeTiZrNi_0.1_	−16.47	12.20	10.85	6.83	1.34
CuFeTiZrNi_0.3_	−18.51	12.82	10.77	6.97	1.25
CuFeTiZrNi_0.5_	−20.14	13.15	10.69	7.11	1.17
CuFeTiZrNi_0.8_	−21.98	13.35	10.58	7.29	1.09
CuFeTiZrNi_1.0_	−22.88	13.38	10.51	7.4	1.05

**Table 3:** The measured hardness values of the CuFeTiZrNi*_x_* alloys and SUS 304.

Alloys	Hardness (HV)	References
SUS 304	207.5 ± 2.7	This work
CoCrFeNi_1.7_Ti_0.3_	318	Hsieh [38]
CuFeTiZrNi_0.1_	934.8 ± 17.0	This work
CuFeTiZrNi_0.3_	921.9 ± 11.5	This work
CuFeTiZrNi_0.5_	914.2 ± 7.7	This work
CuFeTiZrNi_0.8_	905.9 ± 14.8	This work
CuFeTiZrNi_1.0_	893.8 ± 17.1	This work

**Table 4:** The electrochemical parameters of the CuFeTiZrNi*_x_* alloys, CuFeTiZr, and SUS 304 in 3.5 wt.% NaCl solution.

Alloys	*i_corr_* (A/cm^2^)	E*_corr_* (V)	References
CuFeTiZr	2.04 × 10^−7^	−0.328	Chen [42]
SUS 304	5.83 × 10^−8^	−0.268	This work
CoCrFeNi_1.7_Ti_0.3_	1.32 × 10^−8^	−0.26	Hsieh [38]
CuFeTiZrNi_0.1_	7.04 × 10^−8^	−0.292	This work
CuFeTiZrNi_0.3_	5.95 × 10^−8^	−0.264	This work
CuFeTiZrNi_0.5_	5.65 × 10^−8^	−0.242	This work
CuFeTiZrNi_0.8_	5.32 × 10^−8^	−0.240	This work
CuFeTiZrNi_1.0_	5.11 × 10^−8^	−0.236	This work

**Table 5:** The average corrosion rates (mm/year) of CuFeTiZrNi*_x_* alloys and SUS 304 in 3.5 wt.% NaCl solution.

Alloys	Corrosion Rate (mm/year)	
	Polarization Test	Immersion Test
CuFeTiZrNi_0.1_	39.17 × 10^−4^	49.25 × 10^−3^
CuFeTiZrNi_0.3_	33.83 × 10^−4^	37.86 × 10^−3^
CuFeTiZrNi_0.5_	26.54 × 10^−4^	14.46 × 10^−3^
CuFeTiZrNi_0.8_	22.07 × 10^−4^	10.08 × 10^−3^
CuFeTiZrNi_1.0_	19.43 × 10^−4^	3.58 × 10^−3^
SUS 304	8.03 × 10^−4^	2.74 × 10^−3^
